# Transsaccadic integration relies on a limited memory resource

**DOI:** 10.1167/jov.21.5.24

**Published:** 2021-05-21

**Authors:** Garry Kong, Lisa M. Kroell, Sebastian Schneegans, David Aagten-Murphy, Paul M. Bays

**Affiliations:** 1 Department of Psychology, University of Cambridge, UK

**Keywords:** eye movements, visual working memory, attention

## Abstract

Saccadic eye movements cause large-scale transformations of the image falling on the retina. Rather than starting visual processing anew after each saccade, the visual system combines post-saccadic information with visual input from before the saccade. Crucially, the relative contribution of each source of information is weighted according to its precision, consistent with principles of optimal integration. We reasoned that, if pre-saccadic input is maintained in a resource-limited store, such as visual working memory, its precision will depend on the number of items stored, as well as their attentional priority. Observers estimated the color of stimuli that changed imperceptibly during a saccade, and we examined where reports fell on the continuum between pre- and post-saccadic values. Bias toward the post-saccadic color increased with the set size of the pre-saccadic display, consistent with an increased weighting of the post-saccadic input as precision of the pre-saccadic representation declined. In a second experiment, we investigated if transsaccadic memory resources are preferentially allocated to attentionally prioritized items. An arrow cue indicated one pre-saccadic item as more likely to be chosen for report. As predicted, valid cues increased response precision and biased responses toward the pre-saccadic color. We conclude that transsaccadic integration relies on a limited memory resource that is flexibly distributed between pre-saccadic stimuli.

## Introduction

Because human visual acuity is highest in the fovea and declines as a function of eccentricity, we frequently move our eyes to bring objects of interest into high-acuity foveal vision (Yarbus, 1967). However, directing our gaze toward one location necessarily means withdrawing it from others. To support detailed and stable scene perception across eye movement-induced displacements, it has been proposed that information from previous fixations can be used to supplement current foveal input in a process known as transsaccadic integration (Irwin & Andrews, 1996).

Because transsaccadic integration relies on information from the recent past to facilitate performance in the present, an intuitive hypothesis is that visual working memory contributes to the process (Aagten-Murphy & Bays, 2019; Irwin, 1991; Prime, Vesia, & Crawford, 2011). Working memory refers to a short-term store capable of maintaining a limited amount of information in an active state to render it available for cognitive processing (Baddeley & Hitch, 1974). The idea that visual working memory could also support perceptual processes is not a new one, as it has already been implicated in resolving ambiguous perception (Kang, Hong, Blake, & Woodman, 2011; Scocchia, Valsecchi, Gegenfurtner, & Triesch, 2013), visual search (Desimone & Duncan, 1995), and sequential stimulus biases (Bliss, Sun, & D'Esposito, 2017; Fritsche, Mostert, & de Lange, 2017).

Pre-saccadic object information maintained in working memory could – with appropriate transformations to account for the retinal shift induced by the saccade (Bays & Husain, 2007; Bridgeman, Van der Heijden, & Velichkovsky, 1994; Burr & Morrone, 2011) – serve as an additional source of information to enhance post-saccadic perception. Previous research suggests that pre- and post-saccadic sources of information are combined according to the principles of probabilistic inference (e.g., Ernst & Banks, 2002), i.e., as a weighted average taking into account the relative reliability of each input (Oostwoud Wijdenes, Marshall, & Bays, 2015). By averaging out independent noise, the resulting integrated percept may exhibit greater precision than either source alone (Ganmor, Landy, & Simoncelli, 2015; Wolf & Schutz, 2015).

Despite its intuitiveness, direct evidence for an involvement of visual working memory in transsaccadic integration is sparse. Several studies have examined the effect of intervening saccades on working memory tasks. Prime, Tsotsos, Keith, and Crawford (2007) observed no difference in a change discrimination task between conditions in which gaze position was maintained or changed between subsequent stimulus presentations, suggesting that saccades by themselves neither impair the operation of visual working memory nor replace it with a separate transsaccadic store. However, two studies using methods sensitive to memory precision (Melcher & Piazza, 2011; Schut, Van der Stoep, Postma, & Van der Stigchel, 2017; Shao et al., 2010) found that making a saccade to a visual item that was irrelevant to the memory task impaired subsequent recall precision for the memory array, with a performance decrement equivalent to increasing the set size of working memory contents by one item (Schut et al., 2017). This suggests that the allocation of memory resources to the saccade target is obligatory. This automatic allocation could be in the service of transsaccadic integration, but is also consistent with the use of visual working memory to facilitate other perceptual or cognitive processes, e.g., to facilitate visual search (Oh & Kim, 2004; Woodman & Luck, 2004) or attentional shifts after the saccade (Hollingworth & Matsukura, 2019).

To date, the most direct evidence supporting an involvement of working memory in transsaccadic integration comes from a study by Stewart and Schütz (2018). As in previous studies, these authors observed transsaccadic performance advantages in estimation of a single stimulus that were close to the predictions based on optimal integration of pre- and post-saccadic input. However, when they placed the same task within the maintenance period of a typical one-item visual working memory task, they found no significant performance benefit over the best individual view of the stimulus (pre- or post-saccadic). In other words, introducing a visual working memory load abolished the evidence for transsaccadic integration. Although this result strongly suggests availability of working memory is important to obtain the benefits of integration, the dual-task design leaves its exact role uncertain. Additionally, the finding that a memory load of one item almost completely abolished transsaccadic integration is unexpected, given the extensive evidence that multiple items can be maintained simultaneously in working memory (see also Melcher, 2009; Melcher & Fracasso, 2012 for evidence that other transsaccadic effects have capacities greater than one).

One of the defining features of visual working memory is that the information it can hold is very limited (Alvarez & Cavanagh, 2004; Cowan, 1998; Luck & Vogel, 1997). In analogue report tasks, this limit manifests as a decline in recall fidelity as the number of items in memory increases (Ma, Husain, & Bays, 2014; Schneegans, Taylor, & Bays, 2020; van den Berg, Shin, Chou, George, & Ma, 2012; Zhang & Luck, 2008). Additionally, working memory allocation is flexible, so resources can be preferentially directed to particular items based on behavioral priority (Bays, 2014; Bays & Husain, 2008; Oberauer & Lin, 2017; Schmidt, Vogel, Woodman, & Luck, 2002; Yoo, Klyszejko, Curtis, & Ma, 2018). In this study, we investigated how the allocation of working memory to pre-saccadic items influences transsaccadic integration. To obtain a sensitive and graded estimate of working memory allocation, we used the relative weighting of pre- and post-saccadic inputs in estimation of an item's color as our main performance measure. Based on previous studies (Ganmor, Landy, & Simoncelli, 2015; Oostwoud Wijdenes et al., 2015; Wolf & Schutz, 2015), we expected this weighting to reflect the relative reliability of pre- and post-saccadic information.

## Experiment 1

Here, we investigated whether transsaccadic integration depends on a limited resource by manipulating pre-saccadic set size. If the role of visual working memory in transsaccadic integration is to store pre-saccadic input, we would expect the quality of the information available for integration to decrease as the number of items in the pre-saccadic image increases. To test this prediction, we presented observers with one to four colored disks in their peripheral vision before prompting them to execute a horizontal saccade past the stimulus array. During the saccade, all but one of the disks disappeared, and the color of the remaining disk changed slightly. Participants were asked to report the color of this disk, and we used the distribution of their responses relative to the pre- and post-saccadic colors to assess the weight assigned to each input. Because the color change was small and occurred while the eye was moving, we expected participants to be mostly unaware of it. We tested this assumption in a structured debriefing following the experiment.

### Methods

#### Participants

Fourteen participants (9 female) aged between 20 and 35 years (mean = 24.7) participated in Experiment 1. Participants reported normal or corrected-to-normal visual acuity. Normal color vision was ensured by a screening test (Ishihara, 1972) performed before the study. Participants were naïve as to the purpose of the experiment and compensated with a payment of £10/hour. The experiments were approved by the Cambridge Psychology Research Ethics Committee. Informed consent was obtained in accordance with the Declaration of Helsinki.

#### Apparatus and stimuli

Stimuli were presented on a 27 inch Asus ROG PG279Q monitor (144 Hz refresh rate, 2560 × 1440 pixels, ULMB mode, and Overclocking disabled) at a viewing distance of 60 cm. The background of the screen was black (0.3 cd/m^2^) throughout the experiment. Eye position was tracked online using a desk-mounted EyeLink 1000 (SR Research). Stimulus generation and presentation was implemented in Matlab using the Psychophysics Toolbox (Kleiner, Brainard, & Pelli, 2007). Custom code used the PC chipset's High Precision Event Timer to synchronize the display and eye tracker, which was sampled asynchronously at 1000 Hz. We measured a mean input lag (defined as the interval between a software request to update the screen and 90% of the desired luminance change completed) of approximately 11 ms, consistent with values previously reported for this display (Fabius, Fracasso, Nijboer, & Van der Stigchel, 2019; Zhang et al., 2018).

#### Design and procedure

**Figure 1.**
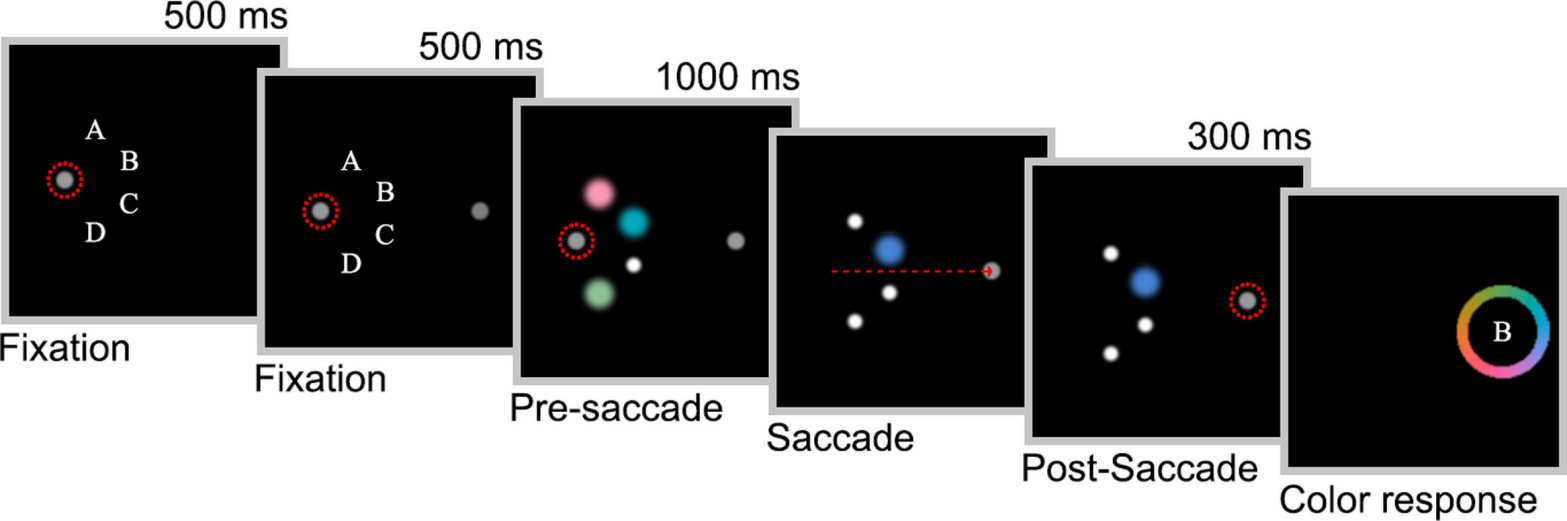
Example trial sequence in Experiment 1 (not to scale), for a trial with set size three. Dashed red circles represent gaze fixations. The dashed red arrow represents the saccade vector. The stimulus changed as soon as gaze crossed the vertical midline of the screen. The color change is exaggerated for illustrative purposes.

The trial sequence is illustrated in Figure 1. Each trial began with the presentation of a gray fixation dot (diameter 0.5 degrees of visual angle, 71.3 cd/m^2^) against a uniformly black background (0.3 cd/m^2^). Depending on saccade direction, the fixation dot appeared 6 degrees to either the left or the right of the screen center. Four letters (A, B, C, and D) were presented at the possible stimulus locations, located on an imaginary circle of 4 degrees radius centered on the fixation dot, at angular positions (–60 degrees, –20 degrees, +20 degrees, and +60 degrees) where 0 degrees is in the horizontal direction toward the center of the display. After fixation had been maintained within 2 degrees of the fixation dot for a period of 500 ms, a second dot (the saccade target) appeared at a horizontal displacement (and hence required saccade amplitude) of 12 degrees from the first fixation point. This point indicated the location to which observers had to saccade once they received the signal. Note that it was not possible to arrange the four stimulus locations to be simultaneously equidistant from both the pre-saccadic and post-saccadic fixation points. We chose to make all four positions equidistant from pre-saccadic fixation, with the result that the A and D positions were further from the post-saccadic fixation point than B and C (10.0 degrees vs. 8.25 degrees).

After 500 ms of further fixation, the letters were replaced by one, two, three, or four colored disks (1 degree in diameter). Colors were randomly drawn from a circle in CIELAB space (L = 74, origin at a = b = 0, radius 40). For set sizes one to three, unoccupied positions were chosen at random, counterbalanced across trials, and filled with gray placeholder dots (0.3 degrees in diameter) to reduce spatial uncertainty. This pre-saccadic display was presented for 1000 ms. After a further 1000 ms, the original fixation dot disappeared and a beep was played at the same time, cueing the participant to make an eye movement to the saccade target as quickly as possible.

Once the gaze crossed the vertical midline of the screen, all but one of the pre-saccadic items (location counterbalanced across trials) were replaced by placeholder dots. The color of the remaining (i.e., post-saccadic) item shifted either clockwise (CW) or counter-clockwise (CCW) by 25 degrees on the color circle. The direction of this shift was chosen randomly. The post-saccadic item was displayed until 300 ms after saccade offset was detected by the eye tracker software.

Immediately after the disappearance of the post-saccadic item, a color wheel (5 degrees in diameter; randomly rotated) appeared around the post-saccadic fixation dot. A letter indicating the position of the post-saccadic item was displayed in the center of the wheel. Participants were instructed to click the color on the wheel that best matched the remembered color of the item indicated by the letter. The letters were used as a non-masking cue to indicate which item to report; although the letter always indicated the item that remained visible after the saccade, pilot testing revealed that participants were often unaware that one of the items was displayed for longer than the other items. When the mouse cursor reached the color wheel, the central letter cue was replaced with a disk (1 degree in diameter) that indicated the color under the current mouse position. After a response was registered, the wheel was replaced by the pre-saccadic fixation dot, initiating the next trial.

A trial was aborted if the gaze deviated more than 2 degrees from the pre-saccadic fixation dot at any time before the saccade, if a saccade had not been initiated by 500 ms after the disappearance of the pre-saccadic fixation dot, if the saccade landed farther than 2.5 degrees from the post-saccadic fixation dot, if the saccade took longer than 150 ms, or if a blink was reported before the color wheel appeared. When a trial was aborted, a feedback message was displayed for 2 seconds in the screen center, and a trial of the same experimental condition was appended to the end of the block.

Observers completed 480 successful trials distributed across four blocks of 120 trials each. Within every block, set size and location of the reported item were randomly interleaved. Each session started with a practice block in which participants were trained on the eye movement component of the experiment. In this practice task, the color report was replaced by feedback on whether the saccade had met all experimental requirements. Error messages were explained verbally by the experimenter when triggered. Practice continued until participants were confident with the oculomotor aspect of the task.

#### Analysis

The primary measures of interest were the bias and dispersion of color responses relative to the pre- and post-saccadic color of the probed item. We estimated these as the circular mean and circular standard deviation (SD), respectively. For this purpose, we rotated and reflected the reported color values, such that 0 degrees corresponded to the pre-saccadic color and positive values were in the direction of the post-saccadic color.

Because responses were reflected on half the trials, any overall CW or CCW response bias was counterbalanced and could not have affected the calculation of the circular mean; however, such a response bias would tend to inflate estimates of the circular SD. To address this, after rotating the responses, but before reflecting them to make the post-saccadic color positive (as described above), we subtracted the overall response bias for each participant, calculated as the circular mean over trials. This operation was applied when estimating circular SD only, but note that it would have no effect on estimates of the circular mean.

Statistical tests of hypotheses were conducted using Bayesian ANOVA and Bayesian *t*-tests in JASP (JASP Team, 2020) with default priors. The outcomes are reported as Bayes factors (BFs). For example, a *t*-test, with a BF_10_ of five indicates that the strength of evidence for a difference is five times greater than the strength of evidence for no difference. Conversely, a BF_01_ of five indicates the same strength of evidence favoring no difference.

### Results and discussion

**Figure 2.**
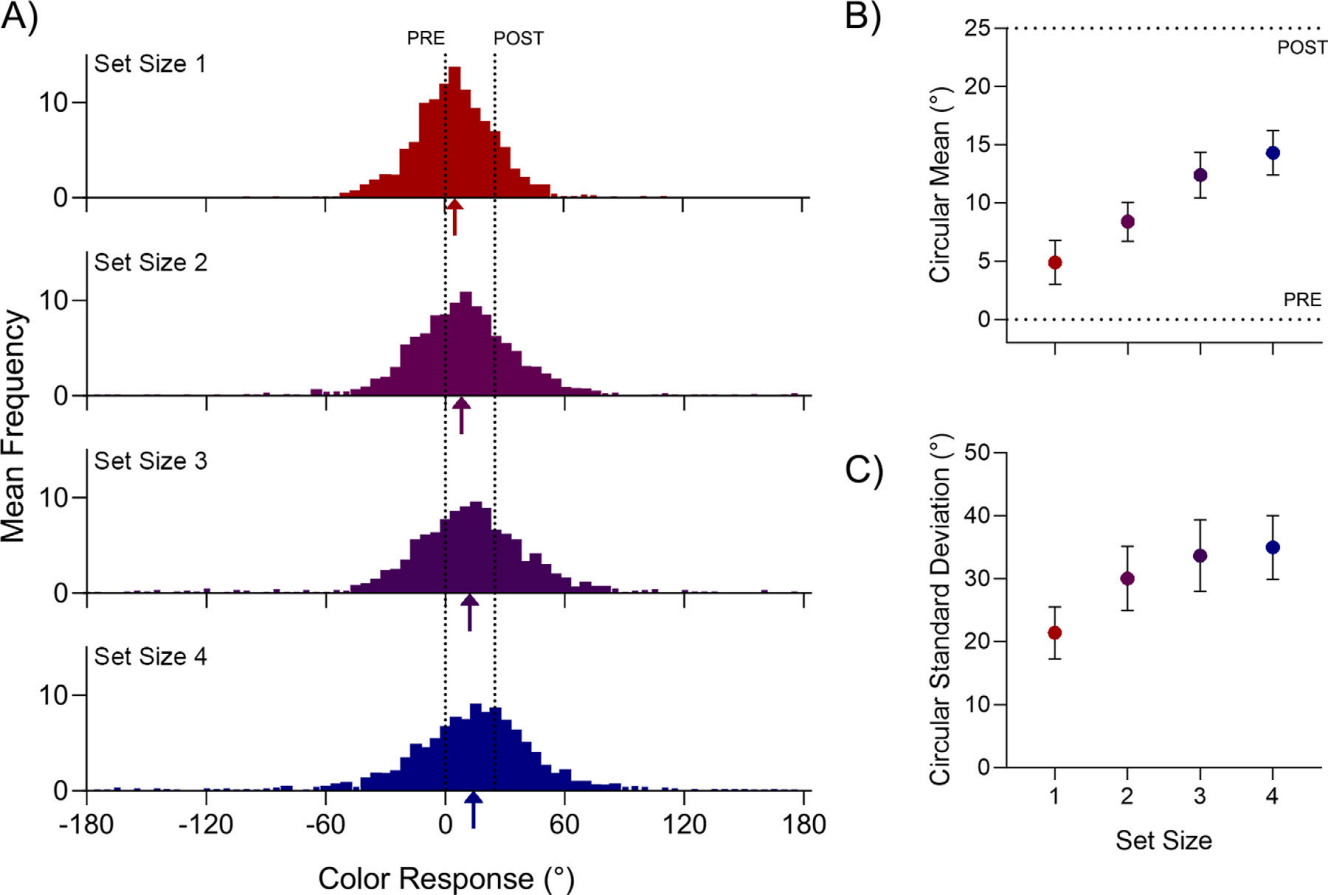
Results of Experiment 1. (**A**) Color response distributions for each set size, plotted with respect to pre- and post-saccadic feature values (indicated by dotted lines). Arrows under the x-axis indicate the circular mean of each distribution. (**B**) Mean circular bias toward the post-saccadic feature as a function of set size. (**C**) Mean circular standard deviation as a function of set size. Error bars denote 95% within subjects confidence intervals (O'Brien & Cousineau, 2014).

The distributions of color responses relative to the pre- and post-saccadic colors are shown for each set size in Figure 2A. We defined bias toward the post-saccadic color value and response variability as the circular mean and circular SD) on a within-observer level (results in Figures 2B, 2C, respectively).

Color estimates were increasingly biased toward the post-saccadic stimulus as set size increased, ranging from 4.90 degrees ± 3.13 degrees (mean ± SD) at set size one to 14.3 degrees ± 5.4 degrees at set size four. A series of Bayesian paired-samples *t*-tests found that bias at set size two exceeded bias at set size one (BF_10_ = 11.7), and bias at set size three in turn exceeded bias at set size two (BF_10_ = 15.1). We obtained weak evidence favoring no difference between set sizes three and four (BF_01_ = 1.30). Likewise, response variability also increased with set size, ranging from 21.9 degrees ± 8.1 degrees at set size one to 37.2 degrees ± 10.6 degrees at set size four. Bayesian paired-samples *t*-tests found that the SD at set size two exceeded the SD at set size one (BF_10_ = 292). The SD at set size three in turn exceeded the SD at set size two (BF_10_ = 5.35). We found weak evidence favoring no difference between set sizes three and four (BF_01_ = 2.76).

To address potential confounds, we investigated whether our results could be influenced by saccadic behavior. Differences in saccade latency could affect pre-saccadic exposure duration, as the color changed only after a saccade was initiated. We found that saccade latencies increased with set size, ranging from 223 ± 19 ms at set size 1 to 251 ± 16 ms at set size four. This effect was supported by Bayesian paired samples *t*-tests, finding shorter latencies from set size one to two (BF_10_ = 96.9), two to three (BF_10_ = 61.1), and three to four (BF_10_ = 7.26). This rules out saccade latency as an alternative explanation for our results: longer saccade latencies imply longer exposure to the pre-saccadic stimulus, which should be associated with a stronger bias toward the pre-saccadic color. Our results show the opposite effect. Furthermore, we found no systematic relationship between saccade latencies and bias (within-subjects Pearson's *r* = 0.015 ± 0.043; Bayesian *t*-test on Fisher-transformed correlations versus no correlation, BF_01_ = 1.82).

The eccentricity of the post-saccadic stimulus relative to the post-saccadic fixation point varied depending on whether it occupied one of the inner or outer array locations. A previous study (Oostwoud Wijdenes et al., 2015) found that biases in integration reflected differences in the relative eccentricity of a stimulus before and after the saccade. Although the eccentricity difference was small, we hypothesized there might be a stronger bias toward the post-saccadic color for items in the inner than outer locations. However, our results did not support a difference in bias (inner = 9.68 degrees; outer = 9.99 degrees; BF_01_ = 3.52), although there was weak evidence for a difference in SD (inner = 30.82 degrees; outer = 33.88 degrees; BF_10_ = 2.06). To confirm this was not contributing to our results, we re-analyzed the main effects of set size with the inclusion of an interaction effect with target location (inner versus outer). We found that the model with the interaction was less likely than the best model without, BF_01_ = 29.82 and 6.52 for bias and SD, respectively.

Although the stimulus locations were chosen to be equidistant from the pre-saccadic fixation point, small differences in gaze direction during the pre-saccadic fixation period could have affected our findings as they determined the retinal eccentricity of the pre-saccadic color array (Oostwoud Wijdenes et al., 2015). We found a general tendency for fixations to be horizontally displaced away from the stimuli as set size increased. Differences in gaze angle ranged from 0.02 degrees ± 0.11 degrees at set size one to 0.07 degrees ± 0.15 degrees at set size four, where positive values denote gaze displacements toward the edge of the screen. Bayesian *t*-tests found weak evidence favoring no difference in horizontal displacements between set sizes one and two (BF_01_ = 1.24) and two and three (BF_01_ = 3.04), but evidence indicating that horizontal displacement at set size four was further from the stimuli than at set size three (BF_10_ = 4.21). Given the magnitudes of the differences in fixation, and that the greatest difference in fixation was between set sizes three and four, where the smallest effect in bias and SD was observed, we feel confident in ruling out variations in pre-saccadic fixation as an explanation for our results. In line with this, we found weak evidence against a correlation between horizontal pre-saccadic fixation displacement and bias in color estimates across all trials (*r* = 0.015 ± 0.052; Bayesian *t*-test on Fisher transformed correlations versus no correlation, BF_01_ = 2.20). Similar analyses of post-saccadic fixation location with respect to the lone post-saccadic stimulus found only evidence against effects of set size (*r* = 0.046 ± 0.042; correlations versus no correlation: BF_01_ = 3.44).

Given the amplitude of the required saccade, strong reproducibility of saccade velocity profiles (e.g., Harwood, Mezey, & Harris, 1999), and low latencies of the eye-tracker and display, we can be confident that the large majority of color changes occurred while the eye was moving. However, we cannot rule out the possibility that some changes occurred before or after the saccade, particularly on trials that were aborted due to aberrant eye movements. To investigate whether any changes had been visible to participants, and whether this could have influenced our results, we performed a structured debriefing after the experiment, which revealed that most participants were unaware of the color change. Four out of 14 participants indicated that they were aware that the color of the disk could change during a trial. Excluding these participants did not change the overall pattern of results. For a formal comparison between participants who reported being aware and unaware of the change, we performed a mixed-effects Bayesian ANOVA, which found no main effect of awareness on either bias (BF_01_ = 1.39) or SD (BF_01_ = 1.81). The model constrained to a main effect of set size was favored over the model, including effects of set size, awareness, and their interaction (for bias: BF = 4.24; for SD: BF = 7.11). Moreover, we performed a permutation test by randomly shuffling aware and unaware labels between participants and computing the difference in bias and SD between the randomly assigned groups. This process was repeated 10,000 times to estimate the expected distribution of difference measures if there was no real difference between aware and unaware participants. We found that the observed difference was greater than the 95th percentile of shuffled data only in one case (for bias at set size 2).

In conclusion, we demonstrate that increasing the number of items presented before the saccade led to a monotonic decrease in the degree to which pre-saccadic information influenced post-saccadic color judgments. Combined with a concurrent increase in response variability, this suggests that the fidelity of pre-saccadic information available for transsaccadic integration declined with set size, consistent with a resource-limited, transsaccadic memory store.

## Experiment 2

A second key property of visual working memory is that resources can be flexibly allocated to stimuli according to their priority for storage. Studies using attentional pre-cues (Bays, 2014; Bays & Husain, 2008; Oberauer & Lin, 2017; Schmidt et al., 2002; Yoo et al., 2018) have demonstrated enhanced recall of items that are visually salient or task-relevant. If pre-saccadic information is held in a resource-limited store, then we would expect that the pre-saccadic color of an item cued before the saccade would similarly be stored with higher fidelity. As a consequence, integrated estimates would be biased more strongly toward the pre-saccadic color. Experiment 2 constitutes a test of this prediction.

### Method

#### Participants

Fifteen participants (11 female) aged between 18 and 34 years (mean = 26.1) participated in Experiment 2.

#### Design and procedure

**Figure 3.**
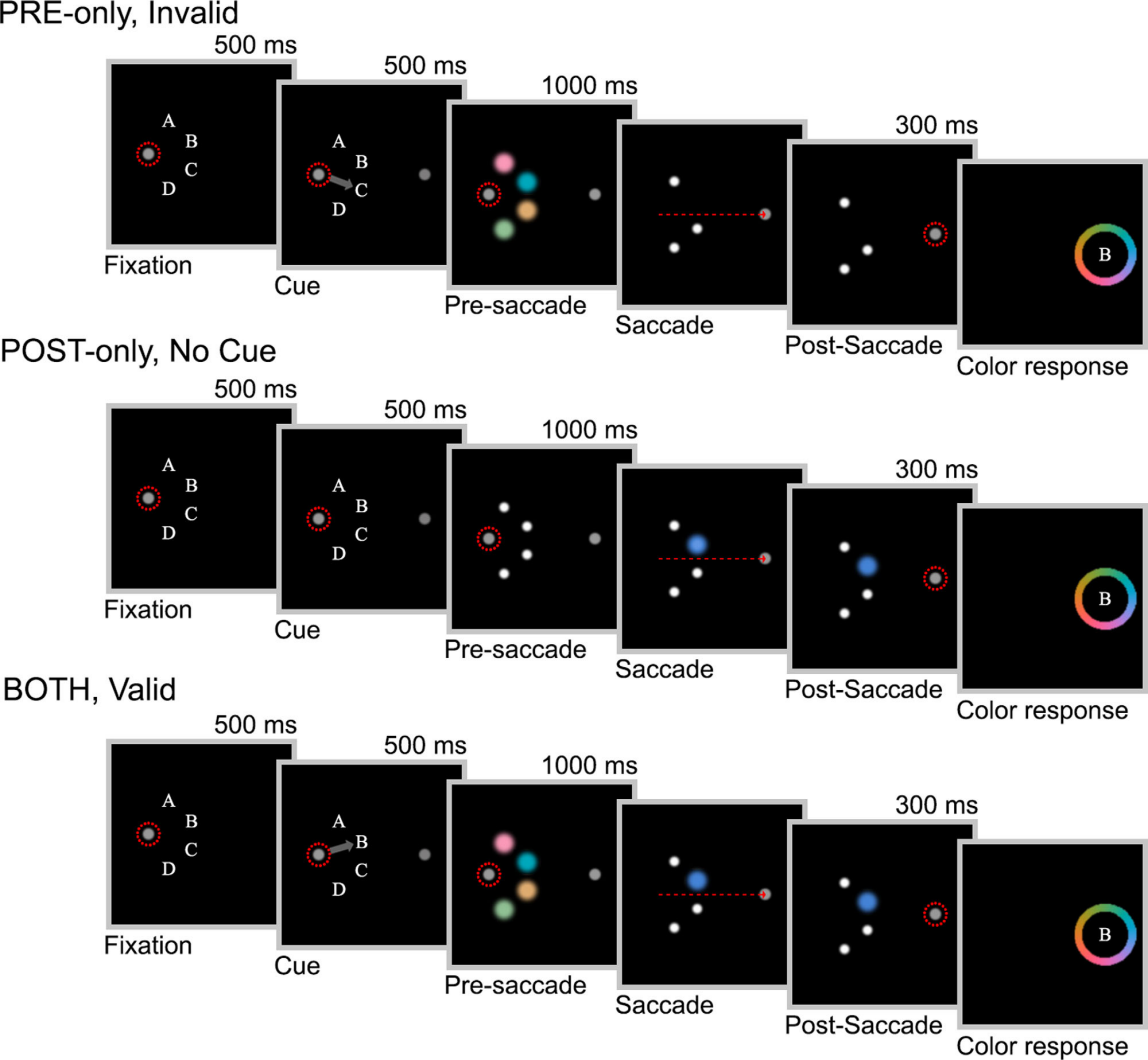
Example trial sequences for Experiment 2 depicting three of the possible nine combinations of conditions (not to scale). Dashed red circles represent gaze fixations. The dashed red arrow represents the saccade vector. The color change was introduced as soon as gaze position crossed the vertical midline of the screen. Color changes are exaggerated for illustrative purposes.

Example trial sequences for Experiment 2 can be seen in Figure 3. The design was identical to the set size four condition in Experiment 1, with the following exceptions: we introduced three cueing conditions of equal frequency: “*valid*,” “*invalid*,” and “*no cue*.” In *valid* and *invalid* conditions, after the initial 500 ms of fixation, a darker grey (54.9 cd/m^2^) arrow cue was presented for 500 ms, pointing to one of the four stimulus locations. The cue did not overlap with the stimulus location, so as to avoid contrast changes around the cued item that could interfere with color processing. On *valid* trials, the item indicated by the cue was subsequently probed (example in Figure 3, bottom panel). On *invalid* trials, one of the other three items was probed with equal probability. Because there were four items and an equal probability of the cue being *valid* or *invalid* in any single trial, the cued item was three times more likely to be probed than any uncued item, providing an incentive to prioritize its storage. Participants were explicitly told about the probabilistic validity of the cue. The *no cue* trials followed the same timings as *valid* and *invalid* trials, with the only difference that no arrow was shown.

The task included trials in which only the pre- or only the post-saccadic stimulus was presented, in addition to trials where both were shown. Trials in which the probed stimulus was presented before and after the saccade, labeled *BOTH* trials (see the example in Figure 3, bottom panel), proceeded in the same way as the set size four trials in Experiment 1. That is, four items were presented before the saccade, only one remained visible after the saccade (the cued item on *valid* trials, an uncued item on *invalid* and *no cue* trials) and that item was indicated for report by the letter corresponding to its location appearing inside a color wheel.

*PRE-only* trials were identical to *BOTH* trials with the exception that the probed item was removed during the saccade (see the example in Figure 3, top panel). No placeholder appeared in its stead to avoid any influence of backward masking. In *POST-only* trials, the pre-saccadic items were replaced with four placeholder dots during the eye movement (see the example in Figure 3, middle panel).

Manipulations of cue validity and presentation time were fully counterbalanced, resulting in a total of nine conditions. Participants completed eight blocks distributed across two experimental sessions of four blocks each. Each block comprised 99 trials (11 per condition, randomly interleaved) amounting to a total of 792 trials. Each session lasted 60 to 90 minutes. Both sessions were performed within a 1-week period. At the beginning of the first session, participants completed an eye movement practice block (see Experiment 1), followed by a practice block of 33 trials involving *no cue* trials only, and a second practice block of 27 trials involving all experimental conditions.

#### Analysis

Bias and variability were calculated in the same way as in Experiment 1. Note that color responses were reflected only for trials in the *BOTH* condition.

### Results and discussion

We expected that cueing a pre-saccadic item would prioritize its processing and enhance the fidelity of the stored memory content. On *valid* trials, this should decrease bias toward the post-saccadic color value and reduce error variability compared to *no cue* trials.

**Figure 4.**
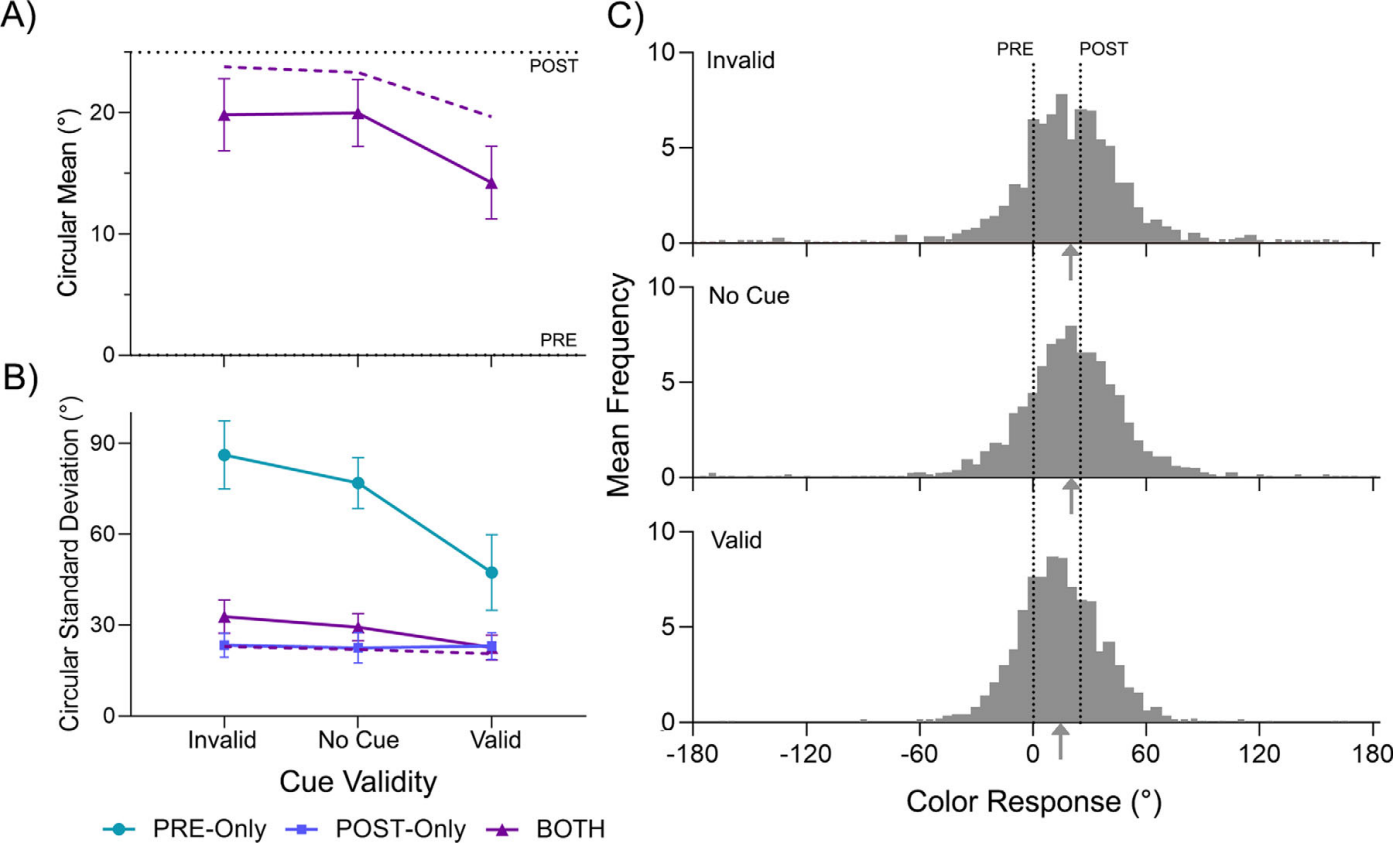
Results of Experiment 2. (**A**) Mean circular bias towards the post-saccadic color in the *BOTH* condition, as a function of cue validity. (**B**) Mean circular standard deviation in each presentation condition, as a function of cue validity. (**C**) Color report distributions in the *BOTH* condition for all cue validities. Arrows under the x-axis indicate the circular mean of the distribution. The purple dashed line denotes model prediction of the *BOTH* condition derived from the *PRE-* and *POST-only* conditions. Error bars denote 95% within subjects confidence intervals (O'Brien & Cousineau, 2014).

To confirm that the cue was effective in modulating behavior, we analyzed the color error variability in the *PRE-only* and *POST-only* conditions. In the *PRE-only* condition (cyan symbols in Figure 4B), the SD was lower on *valid* trials (47.4 degrees ± 25.4 degrees) than on *no cue* trials (77.0 degrees ± 19.7 degrees; BF_10_ = 212). The SD on *invalid* trials was on average higher (86.2 degrees ± 24.6 degrees), although there was only weak evidence for a difference from *no cue* trials (BF_10_ = 2.05). In the *POST-only* condition, as shown by the blue symbols in Figure 4B, no difference in SD was found between cue conditions (repeated-measured Bayesian ANOVA, BF_01_ = 4.44). This pattern of results confirms that the cueing manipulation was effective in modulating the fidelity of the pre-saccadic representation of the cued item, but did not influence post-saccadic processing.

We next examined the effect of cues on integration in the *BOTH* condition. Color estimates on *valid* trials were less biased toward the post-saccadic color than on *no cue* trials (14.2 degrees ± 4.0 degrees vs. 20.0 degrees ± 3.4 degrees; BF_10_ = 1.49 × 10^5^), consistent with a reliability-based increase in weighting of the pre-saccadic color of the cued item. Color bias on *invalid* trials (19.9 degrees ± 3.90 degrees) did not differ from *no cue* trials (BF_01_ = 3.79). This is broadly consistent with the weak effect of invalid cues on variability observed in the *PRE-only* condition.

The flexible resource account predicts that the increase in fidelity for a cued item should be matched by a decrease in fidelity for uncued items, and this has been observed in previous studies of visual working memory (e.g., Bays, Gorgoraptis, Wee, Marshall, & Husain, 2011; Gorgoraptis, Catalao, Bays, & Husain, 2011). The failure to find a clear *invalid* cue effect in the present study may reflect the fact that, whereas the benefit of a valid cue accrues only to the cued item, the corresponding cost of an invalid cue is distributed between all uncued items (3 in this case). As a consequence, the expected effects of *invalid* cues are smaller and more difficult to detect than those of *valid* cues.

The variability estimates for the *BOTH* condition are shown by purple symbols in Figure 4B. The SD on *valid* trials (22.6 degrees ± 3.9 degrees) was lower than the SD on *no cue* trials (29.3 degrees ± 6.5 degrees; BF_10_ = 35.63). Numerically, the SD on *invalid* trials exceeded the SD on *no cue* trials (32.8 degrees ± 9.1 degrees), although evidence for this difference was only weak (BF_10_ = 2.43). Variability in *BOTH* trials was lower than variability in *PRE-only* trials in every cueing condition (lowest BF_10_ = 22.4). However, we did not find a consistent decrease in variability compared to the *POST-only* condition: for *invalid* (BF_10_ = 26.6) and *no cue* (BF_10_ = 13.5) trials, the *BOTH* condition showed a higher SD, whereas there was evidence for no difference in the *valid* condition (BF_01_ = 3.55).

Given that the SD in the *BOTH* condition was higher than in the *POST*-*only* condition, it is clear that performance did not demonstrate the benefit predicted by optimal integration, in contrast to some previous studies testing a single stimulus (Ganmor et al., 2015; Wolf & Schutz, 2015). The purple dashed line in Figures 4A and 4B correspond to predicted performance based on an optimally weighted average of *PRE*- and *POST-only* data. While the effects of cue condition were as expected (decreased bias toward post-saccadic color and decreased SD for *valid* cues) and qualitatively matched the empirical data from the *BOTH* condition, the optimal integration model consistently overestimated the bias toward post-saccadic color and underestimated the SD. We will consider possible explanations for this in the General Discussion.

As in Experiment 1, the eccentricity of the post-saccadic stimulus relative to the post-saccadic fixation point varied depending on whether it occupied one of the inner or outer array locations, possibly leading to a stronger bias toward the post-saccadic color for items in the inner than outer locations. Again, our results did not support a difference in bias (inner = 17.21 degrees; outer = 18.61 degrees°; BF_10_ = 1.01), although there is now stronger evidence for a difference in SD (inner = 26.39 degrees; outer = 30.58 degrees; BF_10_ = 5.72). To confirm this was not contributing to our results, we re-analyzed the main effects of set size with the inclusion of an interaction effect with target location (inner versus outer). We found that the model with the interaction was less likely than the best model without, BF_01_ = 5.44 and 1.86 for bias and SD, respectively.

As in Experiment 1, we tested whether awareness of the intrasaccadic color change, as assessed in a structured debriefing, affected results. Nine out of 15 participants indicated that they were aware that the color had changed on some trials. Excluding these participants did not alter the overall pattern of results. For a formal comparison between participants who reported being aware and unaware of the change, we performed a mixed-effects Bayesian ANOVA on the trials from the *BOTH* condition, which favored no main effect of awareness in either the bias (BF_01_ = 2.26) or SD (BF_01_ = 2.25). A model restricted to an effect of cue validity was more likely than a model including effects of cue validity, awareness, and an interaction, both for bias (BF = 5.27) and SD (BF = 5.80). We furthermore performed a permutation test by randomly shuffling awareness labels (see Experiment 1). The observed difference was found to be within than the 95^th^ percentile of the shuffled data in all cueing conditions in the *BOTH* trials.

## General discussion

The movement of the eyes is key to being able to see the world clearly (Yarbus, 1967). These eye movements compensate for the limited resolution of human peripheral vision by supporting the accumulation of relevant visual information across successive fixations (Irwin & Andrews, 1996). Across two experiments, we investigated the nature of representation of the pre-saccadic input available for integration. In Experiment 1, we found that increasing the number of pre-saccadic items monotonically decreased the relative weighting of the pre-saccadic representation in the integrated estimate, consistent with a decline in fidelity of the pre-saccadic representation. In Experiment 2, we found that a valid pre-cue highlighting one item in the pre-saccadic display led to an increase in the relative weighting of the pre-saccadic representation in integration, compared to a condition with no cue. This suggests that the prioritized item was represented with enhanced fidelity in the pre-saccadic store. Both the decline in fidelity with set size (Bays & Husain, 2008; Schneegans et al., 2020; van den Berg et al., 2012; Zhang & Luck, 2008) and the flexibility in allocation (Gorgoraptis et al., 2011; Oberauer & Lin, 2017; Schmidt et al., 2002; Yoo et al., 2018) are characteristic qualities of visual working memory, often theorized to be the storage medium underlying transsaccadic integration (see Aagten-Murphy & Bays, 2019 for a review).

The principle underlying optimal integration is that a correctly weighted average of two (or more) sources of information can have lower variability than either source alone. Consistent with this, some previous studies of transsaccadic integration have observed an advantage in estimating a visual feature viewed both before and after a saccade, compared to either one of the two views on its own (Ganmor et al., 2015; Stewart & Schütz, 2018; Wolf & Schutz, 2015). In Experiment 2 we found variability in the *BOTH* condition was consistently lower than the *PRE-only* condition but we did not see a similar benefit in comparison to the *POST-only* condition.

There are a number of methodological differences between the previous studies that observed an integration benefit and our Experiment 2, e.g., the location of the stimuli (fovea or periphery), the duration of pre- and post-saccadic exposures, and the presence of an intrasaccadic feature change (color shift). However, we would argue that the most likely basis for the discrepancy is that the previous results were all based on a single stimulus present both before and after the saccade, whereas our pre-saccadic displays contained four items. The predicted precision advantage for *BOTH* over *POST-only* conditions rely on the assumption that variability observed in the *POST-only* condition is an accurate measure of the variability of the post-saccadic representation in the *BOTH* condition. Only one item was presented on each trial in the *POST-only* condition. Three of the four items presented in the *BOTH* condition were removed during the eye movement, and in principle the pre-saccadic representations of those items could be dropped from memory as soon as the saccade landed, leaving an effective set size of one after the saccade.

However, removal of stimuli from working memory takes time (Williams & Woodman, 2012), so it is likely that the item still visible after the saccade in fact faced continuing competition for representation from the other three items. Additionally, it is possible that replacement of the non-target colors with white placeholders, despite occurring during the saccade, exogenously drew attentional resources away from the target, which underwent a much smaller change. As a result of either or both of these possibilities, the *POST-only* condition, with no competing representations, would have underestimated the true variability of the post-saccadic representation in the *BOTH* condition, explaining why we did not see the expected advantage due to integration.

Further evidence that the *POST-only* condition underestimated variability can be seen in the observed bias in the *BOTH* condition. If the differences in pre- and post-saccadic reliability were truly as great as indicated by *PRE-* and *POST-only* conditions, then we would expect estimates to be based almost entirely on post-saccadic information. Instead, the observed biases indicated a substantial influence of the pre-saccadic color on responses. An additional prediction is that the effect of lingering pre-saccadic representations in the *BOTH* condition should be least in the *valid* cueing condition, because in that condition pre-saccadic memory resources should have been allocated preferentially to the one item that was still present after the saccade. Broadly in agreement with this prediction, the *valid* condition was the only one in which mean variability in the *BOTH* condition was numerically lower than the *POST-only* condition (although a *t*-test found moderate evidence against a difference).

Our results suggest that transsaccadic integration of an object's features requires allocation of limited working memory resources to that object before the saccade. It is tempting to think that this resource should be fully dedicated to the saccade target, as this is usually the most goal-relevant object in everyday scenarios. Indeed, performance costs on an unrelated memory task due to a saccade (Schut et al., 2017; Shao et al., 2010), equivalent to one extra memory item, have been explained as obligatory allocation of working memory to the saccade target before the eye movement. However, the present results demonstrate that the visual system has the ability to integrate items other than the saccade target. In fact, as the saccade target is foveated after the saccade, its post-saccadic representation will typically be very reliable, meaning that there is little benefit to integration. In contrast, non-target items may benefit more from integration, as their peripheral location leads to a less precise post-saccadic representation. On the other hand, the brevity of fixations in natural vision may limit the ability to encode peripheral details of a scene for the purposes of integration. Our experiments had a pre-saccadic encoding duration of one second, longer than typical fixations in natural viewing, although we note that other studies have demonstrated integration of stimuli besides the saccade target at shorter encoding durations (e.g., Oostwoud Wijdenes et al., 2015; Stewart & Schütz, 2019).

In summary, we found evidence that transsaccadic integration relies on a limited resource for representation of pre-saccadic input, and that this resource can be flexibly allocated to prioritize goal-relevant objects. A parsimonious account of these findings, consistent with results from dual task studies, is that visual working memory is the medium through which pre-saccadic information contributes to post-saccadic perception.
